# Topography of mutational signatures in non-small cell lung cancer: emerging concepts, clinical applications, and limitations

**DOI:** 10.1093/oncolo/oyae091

**Published:** 2024-06-22

**Authors:** Ritujith Jayakrishnan, David J Kwiatkowski, Michal G Rose, Amin H Nassar

**Affiliations:** Department of Internal Medicine, Yale School of Medicine, New Haven, CT, United States; Department of Pulmonary Medicine, Brigham and Women’s Hospital, Boston, MA, 02115, United States; Yale University School of Medicine and Cancer Center, Veterans Affairs Connecticut Healthcare System, West Haven, CT 06516, United States; Department of Medicine, Medical Oncology Division, Yale Cancer Center, New Haven, CT, United States; Yale University School of Medicine and Cancer Center, Veterans Affairs Connecticut Healthcare System, West Haven, CT 06516, United States

**Keywords:** non-small cell lung cancer, mutational signatures, DNA damage, mutation

## Abstract

The genome of a cell is continuously battered by a plethora of exogenous and endogenous processes that can lead to damaged DNA. Repair mechanisms correct this damage most of the time, but failure to do so leaves mutations. Mutations do not occur in random manner, but rather typically follow a more or less specific pattern due to known or imputed mutational processes. Mutational signature analysis is the process by which the predominant mutational process can be inferred for a cancer and can be used in several contexts to study both the genesis of cancer and its response to therapy. Recent pan-cancer genomic efforts such as “The Cancer Genome Atlas” have identified numerous mutational signatures that can be categorized into single base substitutions, doublet base substitutions, or small insertions/deletions. Understanding these mutational signatures as they occur in non-small lung cancer could improve efforts at prevention, predict treatment response to personalized treatments, and guide the development of therapies targeting tumor evolution. For non-small cell lung cancer, several mutational signatures have been identified that correlate with exposures such as tobacco smoking and radon and can also reflect endogenous processes such as aging, APOBEC activity, and loss of mismatch repair. Herein, we provide an overview of the current knowledge of mutational signatures in non-small lung cancer.

## Teaser

This article provides an overview of the current knowledge of mutational signatures in non-small lung cancer.

**Implications for practice**

Mutational signatures are patterns of somatic mutations that have emerged from the sequencing of numerous human cancer genomes. In turn, mutational signatures have prognostic, diagnostic, and therapeutic potential. This review synthesizes all of the above within the genetic landscape of non-small cell cancer. It highlights signatures associated with exogenous influences like tobacco and radon or endogenous influences like DNA-mismatch repair. It describes the ability of mutational signatures to improve screening for exposures, diagnosis of cancer, and development of personalized treatments. With the increasing prevalence of genetic testing, mutational signatures are poised to gain more clinical applicability, underscoring the need for further research in this field.

## Introduction

Lung cancer remains the leading cause of cancer-related deaths in both men and women in the US.^1^ It is estimated that in 2023 in the US, there will be over 200 000 new cases of lung cancer and approximately 130 000 deaths.^1^ Although treatment breakthroughs have improved survival rates, the mortality rate remains high, due in large part to diagnosis in most patients when the disease has spread to the mediastinal lymph nodes or distant sites. The 5-year survival for localized disease is roughly 63% whereas for distant disease it is 8%.^2,3^ In addition, although targeted therapies can be very effective for lung cancer, the majority of patients do not have a mutation enabling a specific targeted treatment.^4-11^

A firm understanding of the molecular underpinnings of the disease is crucial. In general, cancer tumorigenesis relies on the accumulation of mutations and is thought to be fueled by driver mutations that confer a growth advantage.^12^ Alterations in genes such as *EGFR*, *KRAS*, *ALK*, *MET*, *HER2, ROS1, and RET* have been shown to promote non-small cell lung cancer (NSCLC) formation and are currently targetable for cancer treatment.^13,14^ However, tumor next generation sequencing now allows us to better understand the passenger mutations as well. These bystander mutations do not provide a clonal growth advantage and are not selected over the evolution of the cancer, but may hold other key information about the disease—they store valuable data regarding a patient’s prior exposures.^15,16^ The mutations and their characteristic patterns, ie, mutational signatures, have been identified by sequencing whole cancer genomes and exomes and have been outlined by several independent studies and international consortiums such as The Cancer Genome Atlas and Pan-Cancer Analysis of Whole Genomes.^17-21^ The study of mutational signatures can help us better understand exposures associated with tumorigenesis, better prognosticate cancers, and guide clinical management. The purpose of this review is to describe the basis of mutational signatures in NSCLC, their significance, their future applications, and their limitations.

## Mutational profiling of non-small cell lung cancer in smokers vs non-smokers

A pivotal juncture in the genomic terrain of non-small cell lung cancer lies in the degree of smoking exposure, highlighting its critical role in shaping the disease’s molecular narrative. Smoking-related lung cancer accounts for the majority of lung cancer diagnoses in the US and displays distinct epidemiological and genomic characteristics compared to non-smoking-related lung cancer.^1,14^ Studies show that the total number of single base substitutions (SBS) is approximately 5-fold higher in smoking-related vs non-smoking-related lung adenocarcinomas, with an average of 12.1 compared to 2.7 (*P* < .000001).^22,23^ Among smokers with lung adenocarcinoma, *KRAS* mutations are more prevalent, affecting 47% of tumors, with a dominance of G12C mutations. These *KRAS* mutations arise from smoking-associated transversions leading to various *KRAS* mutations (G12C, G12V, G12A, and G12R) and transitions resulting in the *KRAS* G12D mutation.^24^

In contrast, non-smoking-related lung cancers are notably enriched for targetable genetic alterations, with 78%-92% of these cancers harboring clinically actionable changes, such as *EGFR* mutations and *ALK* fusions.^22,25^ Furthermore, non-smoking-related lung cancers exhibit a significantly lower tumor mutational burden (TMB) than their smoking-related counterparts, being 7-fold lower, and show a depletion of smoking-related genetic signatures; only 6% contain such signatures, which may reflect secondhand smoke exposure.^26^

## Mutational signatures—mutation patterns due to distinct mutation processes

**Figure 1.**
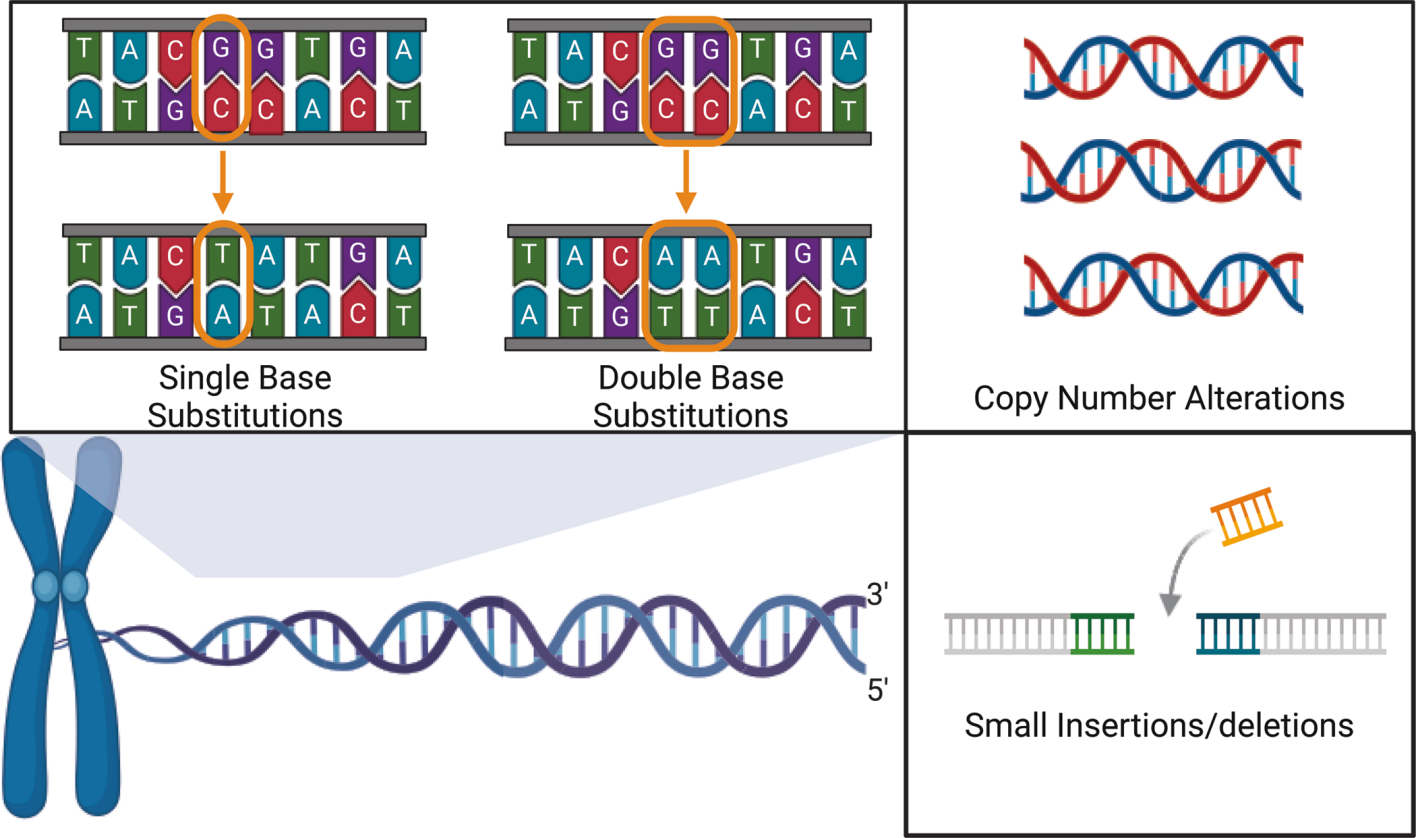
Mutation signature categories. Shown are single and double-base substitutions, small insertions/deletions, and copy number alterations.

Mutation signatures are consistent patterns of mutations that can be recognized as recurrent mutations occurring in specific DNA sequence contexts. Mutational signatures occur because different DNA damaging agents (eg, tobacco smoke carcinogens), endogenous errors in DNA repair, or endogenous processes (eg, high expression of APOBEC enzymes) cause different types of mutation.^27^ Non-negative matrix factorization (NMF) is an algorithm that has been used to decipher these signatures from mutations identified in cancer genomes.^28^ Mutational signatures can be characterized as SBS, double-base substitutions (DBS), small insertions-deletions (ID), copy number alterations, and structural variants (Figure 1). These have been extensively studied and documented in the Catalogue of Somatic Mutations in Cancer (COSMIC) database.^29^

**Figure 2.**
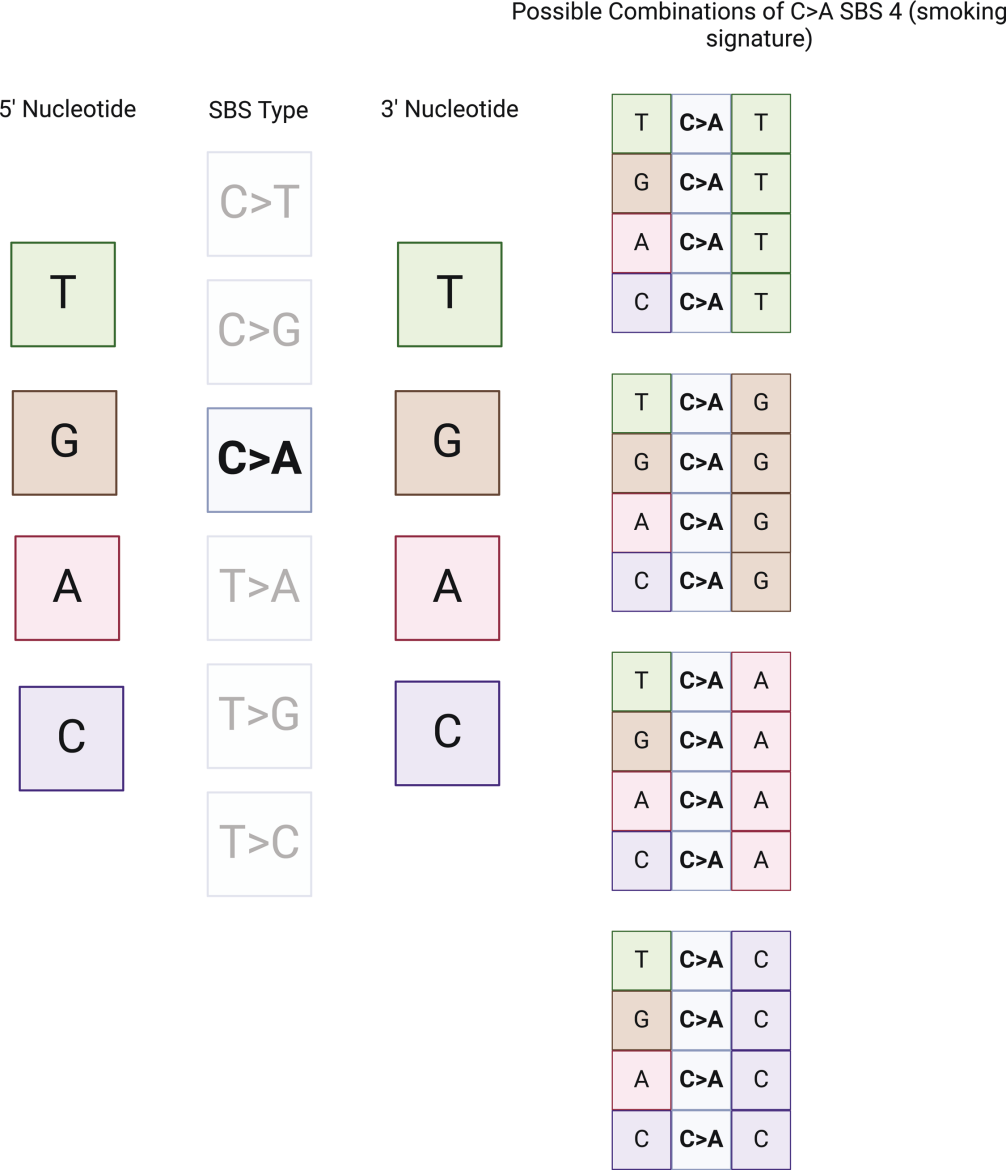
SBS 4 mutation signature associated with tobacco smoking. This is a C > A substitution which can occur in all sequence contexts, meaning both the preceding nucleotide and the following nucleotide can be any nucleotide.

SBS are characterized by not only the mutated base but also the bases adjacent to it. This creates a total of 96 combinations (6 × 4 × 4) – 6 different possible single base substitutions (C > A, C > G, C > T, T > A, T > C, T > G), each of which could be flanked with each of the 4 bases on each of the 5ʹ side and 3ʹ side. For example, T > A mutation may occur in the following contexts: ATA, ATC, ATG, ATT, etc. It has also been observed that certain mutations patterns occur predominantly on transcribed or the untranscribed strand. For example, a C > A substitution on the transcribed strand is the same as a G > T substitution on the untranscribed strand and the mutational distribution (eg, C > A vs. G > T) on the transcribed versus untranscribed strands determines whether strand bias exists. As such, introduction of strand bias to mutational signature analyses extended the previously defined 96 substitution categories to 192 categories (Figure 2).^30^ This is of particular importance as certain mutational signatures such as UV-light have a transcriptional strand bias.^30^

Studies dating back over 35 years first identified patterns of DNA damage caused by tobacco carcinogens (C > A transversions) and by UV-light (C > T and CC > TT transitions).^31,32^ However, systematic analysis of mutational signatures did not occur until 2012 when whole-genome sequencing of 21 breast cancers led to identification of recurrent SBS patterns that were consolidated into 5 mutational signatures.^28,33^ This study also highlighted the presence of regional hypermutation or clustering of mutations in small chromosomal regions, referred to as *kataegis* (from ancient Greek, meaning shower or thunderstorm). Continuing investigation using data from larger cancer genome analyses have identified 2 related phenomena: *omikli* (ancient Greek “fog”), referring to diffuse genome-wide hypermutation, vs *kataegis,* with hypermutation restricted to chromosomal regions.^34^ Much larger cancer genome data sets, analyzed using NMF, have led to refined definition of mutational signatures such that there are now more than 60 SBS signatures from analysis of 23 829 cancer cases (see COSMIC web page https://cancer.sanger.ac.uk/signatures/sbs/), 19 DBS signatures, and 23 insertion-deletion signatures.^35^ This is regularly being updated on the COSMIC website.

## Mutational signatures in lung cancer

**Figure 3.**
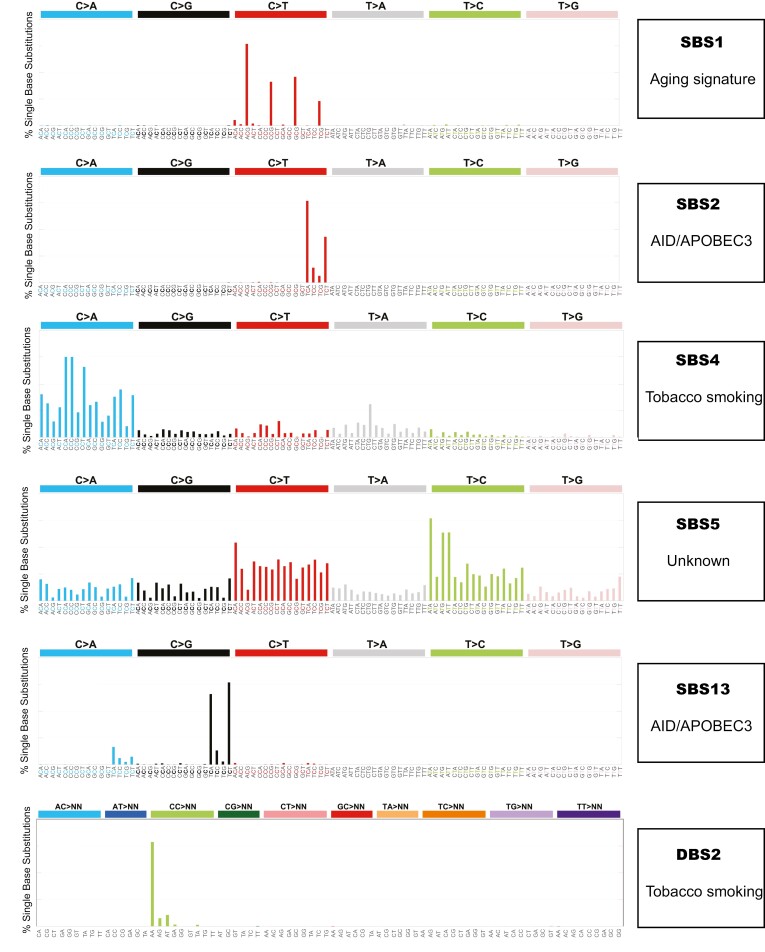
Most common mutation signatures in non-small cell lung cancer. More than one mutation signature can be seen in a patient’s tumor.

A study published as part of Pan-Cancer Analysis of Whole Genomes Consortium consolidated mutational signatures per cancer type and identified at least 6 common mutation signatures in lung cancer (shown in Table 1 and Figure 3).^35^ Other less frequent signatures in lung cancer include defective DNA repair, defective mismatch DNA repair, improper DNA polymerase activity, reactive oxygen species, or prior chemotherapy treatment. Importantly, a single tumor typically harbors more than one mutation signature with a mode of 3 concomitant mutation signatures.^35^

**Table 1.** Table of common mutational signatures in non-small cell lung cancer

Mutational signature	Primary type of substitution	Related to	Transcription strand bias?	Most common sites of mutation
SBS1	C > T transitions	Aging	No	Deamination of 5-methylcytosine
SBS2	C > T transitions	AID/APOBEC3	No	TCN
SBS4	C > A transversions	Tobacco smoking	Yes (transcribed strand)	Unclear
SBS13	C > G transversions	AID/APOBEC	No	TCN
DBS2	CC > AA	Tobacco smoking	Yes (transcribed strand)	Unclear
ID3	1 base-pair deletion of C	Tobacco smoking	Yes (untranscribed strand)	Short cytosine mononucleotide repeats (5 or less)

## Exogenous influences

### SBS4: cigarette smoke

Approximately 80% of lung cancer cases globally are associated with the carcinogens related to the combustion of tobacco, either as firsthand or secondhand smoke.^36^ Tobacco smoke contains over 60 carcinogens, primarily polycyclic aromatic hydrocarbons (PAH) and acrolein, and is known to create bulky adducts at purine bases.^31,37,38^ This distorts the DNA helix and predisposes to C > A transversions which is the basis for the SBS4 signature (Figure 2).^22^ This signature has been reproduced in vitro by exposing cells to benzo[a]pyrene, a PAH.^39^

*TP53* is one of the most commonly mutated genes in cigarette-smoke-related lung cancer, and mutation events in current or former smokers typically fit this pattern.^40^ Of particular significance in this signature is a bias in the location of mutations—they tend to occur most frequently at methylated CpG dinucleotides and preferentially on the transcribed strand.^34,41,42^ While commonly seen in lung cancers, this signature is less evident in other tobacco-associated cancers like bladder, esophageal, or some head and neck cancers, indicating a tumor-specific microenvironment effect and/or improved clearance at the other sites.^22,30^ This signature has also been seen in 6% of non-smokers, although it is assumed to be in the setting of exposure to passive smoke or misrepresentation of smoking habits.^22,25^ A retrospective study of 76 patients with NSCLC and high TMB found SBS4 in 33% of patients.^43^

Furthermore, tumor samples from smokers tend to have higher TMB, which refers to the number of non-synonymous mutations per megabase sequenced. In fact, there is a statistically significant positive correlation between number of base substitutions and pack years smoked for all cancer types.^22^

Two other signatures are commonly seen in lung cancers with predominant SBS4 signature: (1) DBS2, CC > AA substitution characterized by transcriptional strand bias with more GG > TT mutations than CC > AA on the untranscribed strands of genes, and (2) ID3, characterized by deletions of cytosine at mononucleotide cytosine repeats. Exposure to tobacco smoke likely underlies all 3 signatures.^35^

### SBS2 and SBS6: radon

Per the environmental protection agency (EPA), radon causes approximately 21 000 lung cancer-associated deaths each year and is the second leading cause of lung cancer after smoking in the US.^44^ A recent study of non-smokers with lung adenocarcinoma in South Korea assessed the impact of radon exposure at home on one’s genetic makeup; it identified increased TMB in patients with high radon exposure compared to patients with low exposure.^45^ Radon is known to decay into alpha particles, which in lung tissues not only induce oxidative stress but also DNA damage, both of which predispose patients to higher TMB.^46^ This not only leads to direct mutagenesis but is also linked to deficiencies in DNA repair and replication, resulting in the production of mutational signatures SBS2 and SBS6.^45^ SBS2, described further below, is a signature produced by an mRNA editing enzyme called APOBEC and is one of the more common mutational signatures in lung cancer while SBS6 has been observed in mismatch repair-deficient tumors.

### Miscellaneous: other exposures with unclear mutational signatures

In vitro studies of exposure of fine particulate matter (2.5 µm or less) from air pollutants have shown APOBEC-related mutational signatures (explained further below) in bronchial epithelial cells.^47^ This pattern was also identified at a higher percentage in individuals with non-smoking-related NSCLC in China, an area with high exposure to these pollutants.^47^

There have been other identified exposures that are mutagenic and promote lung cancer, including asbestos, arsenic, cooking oil vapor, indoor coal burning, and infections.^48^ However, no clear mutational signatures have been identified for them so far. This is a field of further promise, with an upcoming study called Sherlock-Lung, hoping to decipher other signatures of significance.^49^ This is crucial given the trends of the disease globally. Many of these above exposures are more prominent outside the US and lead to a higher proportion of cancers—never smokers in China, eg, have more lung cancers attributable to tuberculosis or household use of coal compared to never smokers in North America or Europe.^50^ Furthermore, current smoking cessation trends will likely lead to an increased proportion of lung cancers arising in never smokers, highlighting the urgency of identifying other risk factors.

## Endogenous influences

The most common mutational signatures that result from errors in DNA replication and repair include SBS1, SBS2, SBS6, and SBS13. SBS2 and SBS13 are both associated with the AID (activation-induced deaminases)/APOBEC enzyme (apolipoprotein B mRNA editing enzyme, catalytic polypeptide) family.^42^ The AID/APOBEC family of cytidine deaminases have functions in both intrinsic and adaptive immunity, initiating hypermutation and class-switching for immunoglobulins and introducing mutations in viruses infecting cells.^51^ However, they also can induce SBS as a response to cytokines produced during an inflammatory response.^52^ Different APOBEC subtypes have slightly modified targets, although all with a similar pattern—they function primarily on the same parent single-stranded nucleic acid strand to deaminate cytosine.^51,53,54^ This has been shown to induce mutagenesis and contribute to carcinogenesis.^55^ Originally identified as one of the mutagens during the first study of mutational signatures, APOBEC in particular is thought to cause *kataegis*.^33^ The clustered mutations seen in *kataegis* are thought to occur during aberrant DNA double-strand repair that produces single-stranded DNA, ideal sites for mutation by the APOBEC enzyme.^51^ Of note, most APOBEC enzymes have a base preference; they often bind to cytosines that are flanked by thymine on the 5ʹ end (TCN where N is any base), with the only exception to this being AID and APOBEC3G.^52^ Recent studies have identified bladder, cervical, head and neck, breast, and lung cancers (adenocarcinoma and squamous cell carcinoma) to be enriched with high levels of APOBEC mutagenesis.^52^ Although SBS2 and SBS13 result from similar mechanisms, SBS2 refers to C > T transitions while SBS13 is a product primarily of C > G transversions.^27^ The study of 76 NSCLC samples referenced above with a high TMB showed that SBS2 and/or SBS13 were seen in approximately 14% of samples.^43^

SBS1, referred to as the clock-like signature, is characterized by C > T transitions at methylated CG (cytosine followed by guanine sequences) dinucleotides.^56^ This pattern of mutagenesis is seen in nearly all cancer types.^34,42^ Studies of this signature have identified an association between patient age at time of cancer diagnosis and the number of mutations seen with this signature—the higher the age at the diagnosis, the more mutations noted.^42^ Hence, these mutations are thought to occur even prior to malignant transformation.^57^ This age-related incidence pattern is also seen in SBS5. The mechanism of SBS5 is unknown, but it is most prominent in cancers due to tobacco exposure.^58^

## Mutational signatures in oncogene-driven lung cancer

The landscape of mutational signatures within oncogene-driven lung cancer has not been fully explored, often due to the limited number of mutations present in such tumors, which restricts the determination of mutation signatures. In a recent study of 44 samples of *ALK*-positive NSCLC, the most commonly seen mutational signature was the clock-like signature (SBS1) whereby C > T transition occurred most frequently, followed by C > A transitions.^59^ In genetically engineered mouse models of lung adenocarcinoma, *EGFR*^*L858R*^-induced lung adenocarcinomas show significant enrichment for C > T transitions compared to *KRAS*-driven NSCLC.^60^ This finding was later corroborated by a separate effort that analyzed 47 EGFR-driven lung adenocarcinoma samples from the Cancer Genome Atlas.^61^

## Mutational signatures as potential guides for treatment and prognosis

Certain mutational signatures are prognostic, and others are associated with varying responses to treatment. For example, in a study of 113 NSCLC samples, the clock-like signature, SBS1, was associated with worse prognosis in NSCLC with lower lymphocyte infiltration and suppressed immune modulation processes.^62^ APOBEC activity has also been shown to be associated with treatment response and resistance to certain agents. In NSCLC patients, elevated levels of APOBEC-related mutational signatures are associated with improvement in progression-free survival and responses to immune checkpoint inhibitors (ICI), even after controlling for factors such as age, smoking status, PD-L1 expression, and MMR status.^63-65^ Additionally, exposure to EGFR, ALK, or KRAS-directed tyrosine kinase inhibitor (TKI) therapy has been shown to induce expression of APOBEC3A (A3A) and APOBEC3B (A3B) in drug-tolerant persistent cells.^66,67^ This induction of A3A mutagenesis was more pronounced in patients who had received multiple lines of TKI therapy, with extended survival. However, the emergence of A3A mutations is not a consistent outcome across all tumors previously treated with TKIs. This variability suggests the existence of additional mechanisms driving the therapy-induced activation of APOBEC enzymes. Furthermore, APOBEC mutagenesis has been associated with chromothripsis and kataegis, phenomena that may explain the occurrence of certain gene fusions observed as resistance mechanisms to EGFR TKIs.^67,68^ In vitro studies have also demonstrated that APOBEC expressing cells are sensitive to Ataxia Telangiectasia and Rad-3 related (ATR) inhibitors, which are currently in clinical trials (eg, NCT04267939).^69^ This, in turn, could hypothetically delay resistance to TKIs for oncogene-driven NSCLC.

## Other clinical applications of mutational signatures

Mutational signatures can be leveraged to improve diagnostic accuracy. In one report, a patient with presumed metastatic NSCLC was found to have signature 7 mutations associated with ultraviolet exposure that have been classically associated with skin cancers after exposure to UV radiation. Further pathological analysis of the specimen prompted reclassification of the patient’s malignancy to metastatic melanoma.^43^ Moreover, mutational signatures offer an alternative approach to identify prior carcinogenic exposures that traditional clinical assessments might overlook, such as exposure to cigarette smoke and radon. Epidemiological studies traditionally categorize individuals into smokers and non-smokers based on self-reported data, which can be unreliable due to recall bias or patient misrepresentation. Therefore, alternative methods are necessary for accurately characterizing prior exposures. In one study, whole-genome sequencing of NSCLC tumor samples detected smoking-related mutational signatures in patients labeled as never-smokers by clinical assessment and suggests that mutational signatures may be a more accurate representation of prior exposures.^58^ Furthermore, in patients identified with a low smoking mutation signature, there may be a need for more thorough investigation for driver mutations, as the prevalence of targetable mutations is often higher in this population.^70^ Mutational signatures coupled with epidemiology studies could also help unveil carcinogenic substances that we are currently unaware of.

## Limitations

The field of mutational signatures is constantly expanding. An analysis of 5000 whole-genome and 20 000 whole-exome sequenced human cancers mapped at least 78 SBS, 11 DBS, and 18 ID mutational signatures.^34,35,71^ The most recent version v3.4 currently is now published on the COSMIC website as mentioned earlier. However, this ever-evolving space still has gaps including mutational signatures that could be attributed to sequencing artifacts. Moreover, many signatures, for example SBS5 listed above, have identified correlations but no clear etiology. Furthermore, previously identified signatures have now had subtypes identified. For example, signature 7 (ultraviolet exposure) now has now has multiple versions, including SBS7a, SBS7b, SBS7c, and SBS7d, which makes the mutational landscape ever more complex.^35^ Furthermore, mutational signatures were created with a tissue-agnostic global approach, one that included all cancers. However, this assumes that all signatures are identical across all tissues and fails to account for the likely possibility that tissue type affects mutagenesis.^72^ Also, there are various techniques used to extract signatures which have not yet been standardized.^73,74^ Additionally, mutational processes that are present at low population frequencies are challenging to identify in a sample. Finally, the tumor genomic landscape often reflects a complex mix of mutational signatures due to multiple exposures. Disentangling and identifying the specific contributions of each signature can pose a significant challenge.

## Conclusions

In lung cancer, various mutational signatures are seen based on prior exogenous or endogenous stressors. The SBS4 pattern is seen consistently in patients with significant cigarette smoke exposure. SBS2 and SBS13, APOBEC-related mutation signatures, are also seen in lung cancer and may be of prognostic and therapeutic value. As sequencing costs diminish, the current paradigm is shifting more toward paired whole-genome sequencing. This will necessitate optimization of existing computational methods to incorporate more genomic alterations such as copy number alterations and structural variants. The expected wealth of genomic, proteomic data, and revolution in microbiome studies will allow the discovery of additional mutational signatures and refinement of the current ones.

## Data Availability

No new data were generated or analyzed in support of this research.

## References

[1] Siegel RL , Miller KD, Wagle NS, Jemal A. Cancer statistics, 2023. CA Cancer J Clin. 2023;73(1):17-48. 10.3322/caac.21763 36633525

[2] Cronin KA , Scott S, Firth AU, et al. Annual report to the nation on the status of cancer, part 1: National cancer statistics. Cancer. 2022;128(24):4251-4284. 10.1002/cncr.34479 36301149 PMC10092838

[3] Schabath MB , Cote ML. Cancer progress and priorities: lung Cancer. Cancer Epidemiol Biomarkers Prev. 2019;28(10):1563-1579. 10.1158/1055-9965.EPI-19-0221 31575553 PMC6777859

[4] Soria JC , Ohe Y, Vansteenkiste J, et al. Osimertinib in untreated EGFR-mutated advanced non-small-cell lung cancer. N Engl J Med. 2018;378(2):113-125. 10.1056/nejmoa1713137 29151359

[5] Herbst RS , Wu YL, John T, et al. Adjuvant osimertinib for resected EGFR-mutated stage IB-IIIA non-small-cell lung cancer: updated results from the phase III randomized ADAURA trial. J Clin Oncol. 2023;41(10):1830-1840. 10.1200/JCO.22.02186 36720083 PMC10082285

[6] Gainor JF , Curigliano G, Kim DW, et al. Pralsetinib for RET fusion-positive non-small-cell lung cancer (ARROW): a multi-cohort, open-label, phase 1/2 study. Lancet Oncol. 2021;22(7):959-969. 10.1016/S1470-2045(21)00247-3 34118197

[7] Gandhi L , Rodríguez-Abreu D, Gadgeel S, et al. Pembrolizumab plus chemotherapy in metastatic non-small-cell lung cancer. N Engl J Med. 2018;378(22):2078-2092. 10.1056/nejmoa1801005 29658856

[8] Reck M , Rodríguez-Abreu D, Robinson AG, et al. Pembrolizumab versus chemotherapy for PD-L1-positive non-small-cell lung cancer. N Engl J Med. 2016;375(19):1823-1833. 10.1056/nejmoa1606774 27718847

[9] Spigel DR , Faivre-Finn C, Gray JE, et al. Five-year survival outcomes from the PACIFIC Trial: durvalumab after chemoradiotherapy in stage III non-small-cell lung cancer. *JCO*. J Clin Oncol: Off J Am Soc Clin Oncol 2022;40(12):1301-1311. 10.1200/JCO.21.01308 PMC9015199 35108059

[10] Langen AJ de , Johnson ML, Mazieres J, et al. Sotorasib versus docetaxel for previously treated non-small-cell lung cancer with KRASG12C mutation: a randomised, open-label, phase 3 trial. Lancet. 2023;401(10378):733-746. 10.1016/S0140-6736(23)00221-0 36764316

[11] Wolf J , Seto T, Han JY, et al. Capmatinib in MET exon 14-mutated or MET-amplified non-small-cell lung cancer. N Engl J Med. 2020;383(10):944-957. 10.1056/nejmoa2002787 32877583

[12] Stratton MR. Exploring the genomes of cancer cells: progress and promise. Science. 2011;331(6024):1553-1558. 10.1126/science.1204040 21436442

[13] Carper MB , Claudio PP. Clinical potential of gene mutations in lung cancer. Clin Transl Med. 2015;4(1):33. 10.1186/s40169-015-0074-1 26603430 PMC4658345

[14] Adib E , Nassar AH, Abou Alaiwi S, et al. Variation in targetable genomic alterations in non-small cell lung cancer by genetic ancestry, sex, smoking history, and histology. Genome Med. 2022;14(1):39. 10.1186/s13073-022-01041-x 35428358 PMC9013075

[15] Stratton MR , Campbell PJ, Futreal PA. The cancer genome. Nature. 2009;458(7239):719-724. 10.1038/nature07943 19360079 PMC2821689

[16] Elliott K , Larsson E. Non-coding driver mutations in human cancer. Nat Rev Cancer. 2021;21(8):500-509. 10.1038/s41568-021-00371-z 34230647

[17] Aaltonen LA , Abascal F, Abeshouse A, et al. Pan-cancer analysis of whole genomes. Nature. 2020;578(7793):82-93. 10.1038/s41586-020-1969-6 32025007 PMC7025898

[18] Hudson TJ , Anderson W, Artez A, et al; International Cancer Genome Consortium. International network of cancer genome projects. Nature. 2010;464(7291):993-998. 10.1038/nature08987 20393554 PMC2902243

[19] Robertson AG , Kim J, Al-Ahmadie H, et al; TCGA Research Network. Comprehensive Molecular characterization of muscle-invasive bladder cancer. Cell. 2017;171(3):540-556.e25. 10.1016/j.cell.2017.09.007 28988769 PMC5687509

[20] Cancer Genome Atlas Research Network. Comprehensive molecular profiling of lung adenocarcinoma. Nature. 2014;511(7511):543-550. 10.1038/nature13385 25079552 PMC4231481

[21] Cancer Genome Atlas Research Network. Comprehensive genomic characterization of squamous cell lung cancers. Nature. 2012;489(7417):519-525. 10.1038/nature11404 22960745 PMC3466113

[22] Alexandrov LB , Ju YS, Haase K, et al. Mutational signatures associated with tobacco smoking in human cancer. Science. 2016;354(6312):618-622. 10.1126/science.aag0299 27811275 PMC6141049

[23] LoPiccolo J , Gusev A, Christiani DC, Jänne PA. Lung cancer in patients who have never smoked—an emerging disease. Nat Rev Clin Oncol. 2024;21(2):121-146. 10.1038/s41571-023-00844-0 38195910 PMC11014425

[24] Dogan S , Shen R, Ang DC, et al. Molecular epidemiology of EGFR and KRAS mutations in 3026 lung adenocarcinomas: higher susceptibility of women to smoking-related KRAS-mutant cancers. Clin Cancer Res. 2012;18(22):6169-6177. 10.1158/1078-0432.CCR-11-3265 23014527 PMC3500422

[25] Devarakonda S , Li Y, Martins Rodrigues F, et al. Genomic profiling of lung adenocarcinoma in never-smokers. J Clin Oncol. 2021;39(33):3747-3758. 10.1200/JCO.21.01691 34591593 PMC8601276

[26] Zhang T , Joubert P, Ansari-Pour N, et al. Genomic and evolutionary classification of lung cancer in never smokers. Nat Genet. 2021;53(9):1348-1359. 10.1038/s41588-021-00920-0 34493867 PMC8432745

[27] Helleday T , Eshtad S, Nik-Zainal S. Mechanisms underlying mutational signatures in human cancers. Nat Rev Genet. 2014;15(9):585-598. 10.1038/nrg3729 24981601 PMC6044419

[28] Alexandrov LB , Nik-Zainal S, Wedge DC, Campbell PJ, Stratton MR. Deciphering signatures of mutational processes operative in human cancer. Cell Rep. 2013;3(1):246-259. 10.1016/j.celrep.2012.12.008 23318258 PMC3588146

[29] Tate JG , Bamford S, Jubb HC, et al. COSMIC: the catalogue of somatic mutations in cancer. Nucleic Acids Res. 2019;47(D1):D941-D947. 10.1093/nar/gky1015 30371878 PMC6323903

[30] Alexandrov LB , Stratton MR. Mutational signatures: the patterns of somatic mutations hidden in cancer genomes. Curr Opin Genet Dev. 2014;24(100):52-60. 10.1016/j.gde.2013.11.014 24657537 PMC3990474

[31] Pfeifer GP , Denissenko MF, Olivier M, et al. Tobacco smoke carcinogens, DNA damage and p53 mutations in smoking-associated cancers. Oncogene. 2002;21(48):7435-7451. 10.1038/sj.onc.1205803 12379884

[32] Pfeifer GP , You YH, Besaratinia A. Mutations induced by ultraviolet light. Mutat Res. 2005;571(1-2):19-31. 10.1016/j.mrfmmm.2004.06.057 15748635

[33] Nik-Zainal S , Alexandrov LB, Wedge DC, et al; Breast Cancer Working Group of the International Cancer Genome Consortium. Mutational processes molding the genomes of 21 breast cancers. Cell. 2012;149(5):979-993. 10.1016/j.cell.2012.04.024 22608084 PMC3414841

[34] Otlu B , Díaz-Gay M, Vermes I, et al. Topography of mutational signatures in human cancer. Cell Rep. 2023;42(8):112930. 10.1016/j.celrep.2023.112930 37540596 PMC10507738

[35] Alexandrov LB , Kim J, Haradhvala NJ, et al; PCAWG Mutational Signatures Working Group. The repertoire of mutational signatures in human cancer. Nature. 2020;578(7793):94-101. 10.1038/s41586-020-1943-3 32025018 PMC7054213

[36] Sun S , Schiller JH, Gazdar AF. Lung cancer in never smokers—a different disease. Nat Rev Cancer. 2007;7(10):778-790. 10.1038/nrc2190 17882278

[37] Munnia A , Giese RW, Polvani S, Galli A, Cellai F, Peluso MEM. Chapter six—bulky DNA adducts, tobacco smoking, genetic susceptibility, and lung cancer risk. In: Makowski GS, ed. Advances in Clinical Chemistry. Vol. 81. Elsevier; 2017:231-277. 10.1016/bs.acc.2017.01.006 28629590

[38] Feng Z , Hu W, Hu Y, Shong TM. Acrolein is a major cigarette-related lung cancer agent: preferential binding at p53 mutational hotspots and inhibition of DNA repair. Proc Natl Acad Sci USA. 2006;103(42):15404-15409. 10.1073/pnas.0607031103 17030796 PMC1592536

[39] Nik-Zainal S , Kucab JE, Morganella S, et al. The genome as a record of environmental exposure. Mutagenesis. 2015;30(6):763-770. 10.1093/mutage/gev073 26443852 PMC4637815

[40] Rodin SN , Rodin AS. Origins and selection of p53 mutations in lung carcinogenesis. Semin Cancer Biol. 2005;15(2):103-112. 10.1016/j.semcancer.2004.08.005 15652455

[41] Rodin SN , Rodin AS. On the origin of p53 G:C --> T:A transversions in lung cancers. Mutat Res. 2002;508(1-2):1-19. 10.1016/s0027-5107(02)00106-9 12379456

[42] Alexandrov LB , Nik-Zainal S, Wedge DC, et al; Australian Pancreatic Cancer Genome Initiative. Signatures of mutational processes in human cancer. Nature. 2013;500(7463):415-421. 10.1038/nature12477 23945592 PMC3776390

[43] van den Heuvel GRM , Kroeze LI, Ligtenberg MJL, et al. Mutational signature analysis in non-small cell lung cancer patients with a high tumor mutational burden. Respir Res. 2021;22(1):302. 10.1186/s12931-021-01871-0 34819052 PMC8611965

[44] US EPA O. Health Risk of Radon. Published August 14, 2014. Accessed October 11, 2023. https://www.epa.gov/radon/health-risk-radon

[45] Lim SM , Choi JW, Hong MH, et al. Indoor radon exposure increases tumor mutation burden in never-smoker patients with lung adenocarcinoma. Lung Cancer. 2019;131:139-146. 10.1016/j.lungcan.2019.04.002 31027691

[46] Hubaux R , Becker-Santos DD, Enfield KS, et al. Arsenic, asbestos and radon: emerging players in lung tumorigenesis. Environ Health. 2012;11(1):89. 10.1186/1476-069X-11-89 23173984 PMC3534001

[47] Fan R , Xu L, Cui B, et al. Genomic characterization revealed PM2.5-associated mutational signatures in lung cancer including activation of APOBEC3B. Environ Sci Technol. 2023;57(17):6854-6864. 10.1021/acs.est.2c08092 37071573

[48] Corrales L , Rosell R, Cardona AF, et al. Lung cancer in never smokers: the role of different risk factors other than tobacco smoking. Crit Rev Oncol Hematol. 2020;148:102895. 10.1016/j.critrevonc.2020.102895 32062313

[49] Landi MT , Synnott NC, Rosenbaum J, et al. Tracing lung cancer risk factors through mutational signatures in never-smokers: the Sherlock-Lung study. Am J Epidemiol. 2021;190(6):962-976. 10.1093/aje/kwaa234 33712835 PMC8316614

[50] Sisti J , Boffetta P. What proportion of lung cancer in never-smokers can be attributed to known risk factors? Int J Cancer. 2012;131(2):265-275. 10.1002/ijc.27477 22322343 PMC3359408

[51] Siriwardena S , Chen K, Bhagwat AS. The functions and malfunctions of AID/APOBEC family deaminases: the known knowns and the known unknowns. Chem Rev. 2016;116(20):12688-12710. 10.1021/acs.chemrev.6b00296 27585283 PMC5528147

[52] Roberts SA , Lawrence MS, Klimczak LJ, et al. An APOBEC cytidine deaminase mutagenesis pattern is widespread in human cancers. Nat Genet. 2013;45(9):970-976. 10.1038/ng.2702 23852170 PMC3789062

[53] Petljak M , Dananberg A, Chu K, et al. Mechanisms of APOBEC3 mutagenesis in human cancer cells. Nature. 2022;607(7920):799-807. 10.1038/s41586-022-04972-y 35859169 PMC9329121

[54] Swanton C , McGranahan N, Starrett GJ, Harris RS. APOBEC enzymes: mutagenic fuel for cancer evolution and heterogeneity. Cancer Discov. 2015;5(7):704-712. 10.1158/2159-8290.CD-15-0344 26091828 PMC4497973

[55] Burns MB , Lackey L, Carpenter MA, et al. APOBEC3B is an enzymatic source of mutation in breast cancer. Nature. 2013;494(7437):366-370. 10.1038/nature11881 23389445 PMC3907282

[56] Lutsenko E , Bhagwat AS. Principal causes of hot spots for cytosine to thymine mutations at sites of cytosine methylation in growing cells. A model, its experimental support and implications. Mutat Res. 1999;437(1):11-20. 10.1016/s1383-5742(99)00065-4 10425387

[57] Alexandrov LB , Jones PH, Wedge DC, et al. Clock-like mutational processes in human somatic cells. Nat Genet. 2015;47(12):1402-1407. 10.1038/ng.3441 26551669 PMC4783858

[58] Ernst SM , Mankor JM, Riet J van, et al. Tobacco smoking-related mutational signatures in classifying smoking-associated and nonsmoking-associated NSCLC. J Thoracic Oncol. 2023;18(4):487-498. 10.1016/j.jtho.2022.11.030 36528243

[59] Liu S , Huang T, Liu M, et al. The genomic characteristics of ALK fusion positive tumors in Chinese NSCLC patients. Front Oncol. 2020;10:726. 10.3389/fonc.2020.00726 32457845 PMC7225306

[60] McFadden DG , Politi K, Bhutkar A, et al. Mutational landscape of EGFR-, MYC-, and Kras-driven genetically engineered mouse models of lung adenocarcinoma. Proc Natl Acad Sci USA. 2016;113(42):E6409-E6417. 10.1073/pnas.1613601113 27702896 PMC5081629

[61] Kim EY , Kim A, Lee G, Lee H, Chang YS. Different mutational characteristics of the subsets of EGFR-tyrosine kinase inhibitor sensitizing mutation-positive lung adenocarcinoma. BMC Cancer. 2018;18(1):1221. 10.1186/s12885-018-5116-9 30522449 PMC6282318

[62] Chong W , Wang Z, Shang L, et al. Association of clock-like mutational signature with immune checkpoint inhibitor outcome in patients with melanoma and NSCLC. Mol Ther Nucleic Acids. 2020;23:89-100. 10.1016/j.omtn.2020.10.033 33335795 PMC7723771

[63] Wang S , Jia M, He Z, Liu XS. APOBEC3B and APOBEC mutational signature as potential predictive markers for immunotherapy response in non-small cell lung cancer. Oncogene. 2018;37(29):3924-3936. 10.1038/s41388-018-0245-9 29695832 PMC6053356

[64] Chen H , Chong W, Teng C, et al. The immune response‐related mutational signatures and driver genes in non‐small‐cell lung cancer. Cancer Sci. 2019;110(8):2348-2356. 10.1111/cas.14113 31222843 PMC6676111

[65] Ravi A , Hellmann MD, Arniella MB, et al. Genomic and transcriptomic analysis of checkpoint blockade response in advanced non-small cell lung cancer. Nat Genet. 2023;55(5):807-819. 10.1038/s41588-023-01355-5 37024582 PMC10181943

[66] Caswell DR , Gui P, Mayekar MK, et al. The role of APOBEC3B in lung tumor evolution and targeted cancer therapy resistance. Nat Genet. 2024;56(1):60-73. 10.1038/s41588-023-01592-8 38049664 PMC10786726

[67] Selenica P , Marra A, Choudhury NJ, et al. APOBEC mutagenesis, kataegis, chromothripsis in EGFR-mutant osimertinib-resistant lung adenocarcinomas. Annals Oncol: Off J Eur Soc Med Oncol. 2022;33(12):1284-1295. 10.1016/j.annonc.2022.09.151 PMC10360454 36089134

[68] Schoenfeld AJ , Chan JM, Kubota D, et al. Tumor analyses reveal squamous transformation and off-target alterations as early resistance mechanisms to first-line osimertinib in EGFR-mutant lung cancer. Clin Cancer Res. 2020;26(11):2654-2663. 10.1158/1078-0432.CCR-19-3563 31911548 PMC7448565

[69] Buisson R , Lawrence MS, Benes CH, Zou L. APOBEC3A and APOBEC3B activities render cancer cells susceptible to ATR inhibition. Cancer Res. 2017;77(17):4567-4578. 10.1158/0008-5472.CAN-16-3389 28698210 PMC5609510

[70] Mack PC , Klein MI, Ayers KL, et al. Targeted next-generation sequencing reveals exceptionally high rates of molecular driver mutations in never-smokers with lung adenocarcinoma. Oncologist. 2022;27(6):476-486. 10.1093/oncolo/oyac035 35298662 PMC9177106

[71] Islam SMA , Díaz-Gay M, Wu Y, et al. Uncovering novel mutational signatures by de novo extraction with SigProfilerExtractor. Cell Genom. 2022;2(11):None. 10.1016/j.xgen.2022.100179 PMC9646490 36388765

[72] Degasperi A , Amarante TD, Czarnecki J, et al. A practical framework and online tool for mutational signature analyses show intertissue variation and driver dependencies. Nat Cancer. 2020;1(2):249-263. 10.1038/s43018-020-0027-5 32118208 PMC7048622

[73] Baez-Ortega A , Gori K. Computational approaches for discovery of mutational signatures in cancer. Brief Bioinform. 2019;20(1):77-88. 10.1093/bib/bbx082 28968631 PMC6357558

[74] Omichessan H , Severi G, Perduca V. Computational tools to detect signatures of mutational processes in DNA from tumours: a review and empirical comparison of performance. PLoS One. 2019;14(9):e0221235. 10.1371/journal.pone.0221235 31513583 PMC6741849

